# Application of 3D Printing in Bone Grafts

**DOI:** 10.3390/cells12060859

**Published:** 2023-03-10

**Authors:** Adam Brachet, Aleksandra Bełżek, Daria Furtak, Zuzanna Geworgjan, Dawid Tulej, Kinga Kulczycka, Robert Karpiński, Marcin Maciejewski, Jacek Baj

**Affiliations:** 1 Student Scientific Group, Department of Forensic Medicine, Medical University of Lublin, ul. Jaczewskiego 9b, 20-090 Lublin, Poland; 2 Student Scientific Group, Department of Anatomy, Medical University of Lublin, ul. Jaczewskiego 4, 20-090 Lublin, Poland; 3 Institute of Health Sciences, John Paul II Catholic University of Lublin, 20-708 Lublin, Poland; 4 Department of Machine Design and Mechatronics, Faculty of Mechanical Engineering, Lublin University of Technology, Nadbystrzycka 36, 20-618 Lublin, Poland; 5 I Department of Psychiatry, Psychotherapy and Early Intervention, Medical University of Lublin, ul. Głuska 1, 20-439 Lublin, Poland; 6 Department of Electronics and Information Technology, Faculty of Electrical Engineering and Computer Science, Lublin University of Technology, 20-618 Lublin, Poland; 7 Department of Anatomy, Medical University of Lublin, ul. Jaczewskiego 4, 20-090 Lublin, Poland

**Keywords:** 3D printing, bone grafts, FDM, scaffold, cells

## Abstract

The application of 3D printing in bone grafts is gaining in importance and is becoming more and more popular. The choice of the method has a direct impact on the preparation of the patient for surgery, the probability of rejection of the transplant, and many other complications. The aim of the article is to discuss methods of bone grafting and to compare these methods. This review of literature is based on a selective literature search of the PubMed and Web of Science databases from 2001 to 2022 using the search terms “bone graft”, “bone transplant”, and “3D printing”. In addition, we also reviewed non-medical literature related to materials used for 3D printing. There are several methods of bone grafting, such as a demineralized bone matrix, cancellous allograft, nonvascular cortical allograft, osteoarticular allograft, osteochondral allograft, vascularized allograft, and an autogenic transplant using a bone substitute. Currently, autogenous grafting, which involves removing the patient’s bone from an area of low aesthetic importance, is referred to as the gold standard. 3D printing enables using a variety of materials. 3D technology is being applied to bone tissue engineering much more often. It allows for the treatment of bone defects thanks to the creation of a porous scaffold with adequate mechanical strength and favorable macro- and microstructures. Bone tissue engineering is an innovative approach that can be used to repair multiple bone defects in the process of transplantation. In this process, biomaterials are a very important factor in supporting regenerative cells and the regeneration of tissue. We have years of research ahead of us; however, it is certain that 3D printing is the future of transplant medicine.

## 1. Introduction

Extensive bone loss caused by high-energy fractures or pathological fractures require bone transplantation. The first documented bone transplant took place in 1686 by a Dutch surgeon, Job van Meekeren, when he used dog cranium (xenograft) to repair a soldier’s skull defect [1]. Today, more than two million bone transplants are performed worldwide each year. The treatment of bone defects of critical size associated with tumors or resulting from trauma remains an unmet clinical need [2,3,4]. Current treatments using xenografts, autografts, or allografts have multiple serious limitations, including limited supply, donor site morbidity, and disease transmission. There is also a risk of foreign body rejection [4,5,6].

Autograft is considered to be the best method, but it is also associated with disorders at the site of collection and poor availability. Allografts (tissues obtained from human cadavers or living donors) and xenografts (tissue that is derived from a species that is different from the recipient of the specimen) carry the risk of excessive immune reaction and, consequently, rejection of the transplant [2].

Reconstruction of the defect in bone tissue as a surgical procedure is a time-consuming and technically difficult process [7]. The intended effect is not always achieved, and the patient is exposed to residual pain, non-union, or a treatment-resistant infection. Then, a decision may be made to perform a secondary amputation [8,9,10,11]. Spatial printing (3D printing) is the process of producing physical objects based on a computer model [12,13,14]. It is a subset of manufacturing called additive manufacturing (AM).

Three-dimensional (3D) additive manufacturing has recently been widely used in a great many medical fields. Among them, orthopedic oncology is the one using it most actively. Bones and their defects and tumors are modeled for surgical planning, personalized surgical tools for personalized surgical instruments, and implant manufacturing, which are currently the most typical applications [15,16,17,18]. 

The beginnings of 3D printing date back to the 1990s. Huge interest in this method results from the detailed control of the entire process, the possibility of creating non-standard shapes, and the production of structures with specific physical properties. Due to lower costs, shorter time, and a wide possibility of adjusting parameters with a precise layer-by-layer method, it is possible to recreate structures on a micro- and macroscopic scale. These methods have also found their application in medicine [19,20,21,22]. Three-dimensional printing technology has revolutionized the medical field in recent years, allowing the creation of custom personalized implants, prosthetics, and surgical instruments [18,20,23,24]. Some of the key applications of 3D printing in medicine include:

- Personalized implants: 3D printing technology can be used to create personalized implants tailored to the specific needs of individual patients. For example, cranial implants can be created to match the exact shape and size of a patient’s skull, providing a more secure and comfortable fit [25,26].
- Prosthetics: 3D printing allows for the creation of custom-fit prosthetics that are lighter, stronger, and more comfortable than traditional prosthetics [27,28].
- Surgical planning and training: Surgeons can use 3D printing to create replicas of a patient’s anatomy, allowing them to plan and practice complex procedures before performing them on a patient [29,30,31,32].
- Surgical tools: 3D printing can be used to create customized surgical instruments, such as scissors, forceps, and retractors. These custom tools can help improve the accuracy and efficiency of surgeries [33,34].
- Tissue engineering: 3D printing is being used to create functional tissue and organs for use in transplants. Researchers are using 3D printing to create structures that mimic the complex architecture of human tissues, with the goal of eventually being able to print functional replacements for damaged or diseased organs [35,36,37,38]. Bioprinting is currently in its early stages, but due to the significant effort put into research real progress is being made [39,40,41]. The technology still has a long way to go, but its possible application will greatly influence the field of transplantology.
- Training and simulation tools: Various devices for students and trainees that are helpful in the process of education. These can include laparoscopy simulators, models of bones, organs, joints, medical equipment, tools, or even entire body parts constructed from various materials with different mechanical properties and colors [42,43,44]. These are easy to replace, modify, and duplicate, and are a great solution for institutions that are not permitted to use actual human specimens.

In the field of orthopedics, it is used inter alia in bone tissue engineering as a substitute for the previously mentioned human bone tissue transplants. This innovative method aims to solve problems related to tissue source, immune rejection, and disease transmission [8], as well as the reconstruction of normal bone structure—compact and spongy bone requires the use of bone scaffolds, cells, and growth factors [45]. The scaffolding method is the most promising and currently most widely studied. The bone scaffold should contain the appropriate density and size of the pores for the proper angiogenesis process [46]. For this purpose, the most common methods are: chemical foaming, solvent casting, and freeze drying [10]. It is possible to adjust the appropriate amount of minerals, such as calcium phosphate, magnesium or silicon. The conducted studies indicate that the presence of these components incorporated into macroporous bone scaffolds has a positive effect on the rate of bone tissue formation and indicates their use in early wound healing [47,48,49].

The porosity of the implanted scaffold holds a critical role in the process of osteogenesis. It improves osteogenesis by protein adsorption and the generation of capillary forces. These forces help to attach cells on the surface of the implant [50]. Moreover, porous structures have significantly increased surface area compared with non-porous structures, which allows for the faster permeation of chemicals, such as drugs and nutrients, aiding in vascularization and metabolite removal [51,52]. Macro–mesoporous composites containing PEEK and mesoporous diopside as bone implants are characterized by in vitro mineralization and cytocompatibility, as well as vascularization potential and osteogenesis in vivo. This is very important in the process of fluid circulation and helps cell migration towards the center of the implant [53,54]. Additionally, porous structures can absorb antibiotics and other substances introduced before implantation [55]. By controlling the porosity of the structure, it is also possible to impact the degradation rate of the scaffold. In the ideal case, the rates of bone regeneration and implant degradation should be equal. For example, mesoporous silica shows beneficial properties as a part of an antibacterial strategy during implantation [56], and can impact the degradation rate of the implant [57]. Nevertheless, too much porosity can negatively impact the mechanical properties and robustness of the implanted structure [58]. This is important, as only strong and stiff materials should be considered for implantation, as they need to provide structural support for the newly formed bone tissues [59,60]. 

**Figure 1:**
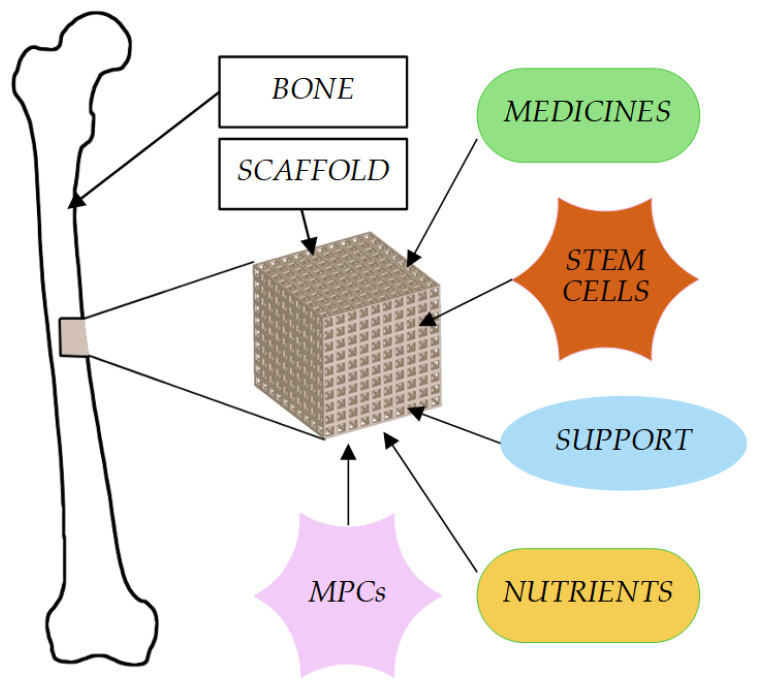
Usage of microporous scaffolds for supporting bone formation.

Scaffolds can be filled with medicines, which are then released locally at the site of scaffold implantation [61] (Figure 1). There is a wide range of materials used in bone tissue engineering, e.g., gypsum, ceramics, resin, and plastics [62,63,64,65,66]. Additionally, the bio-ink ensures the proper regeneration and reconstruction of bone tissue. The resulting material largely corresponds to the morphological features of normal human tissue [67]. The advantage of the entire process is the ability to adjust all parameters to the patient’s needs and, consequently, to improve the condition of life [45].

## 2. Bone Grafts

Bone transplantation is a medical procedure aimed at filling the defects in the recipient’s bone by implanting the donor’s bone. Several methods of this procedure have been described [68,69]. The first is demineralized bone matrix (DBM), which consists of taking the bone matrix from a donor and treating it with chemicals—acids—and then demineralizing it. Furthermore, additional substances are used, such as ethylene oxide, which kills pathogens but affects the acceptance of the transplant by the recipient’s organism, meaning that it is practically not used. Currently, gamma irradiation is more commonly used because, in addition to neutralizing pathogens such as HIV or HCV viruses, it has less impact on graft acceptance; however, it weakens the structural integrity of the graft material. Currently, DBM is used to fill cavernous defects and to fill in non-fused bones [70]. Similar to DBM, a cancellous allograft is used, but they differ in the method of fixation [71]. Unlike the demineralized bone matrix, it does not undergo demineralization but is deeply frozen. This increases the possibility of contracting HIV; however, the chance of it is still low. Another method of bone transplantation is the nonvascular cortical allograft. This bone has a much higher density than the trabecular bone, making it more difficult to eliminate pathogens, and therefore there is a risk of HIV infection from the donor. This material is freeze-dried, which reduces its immunogenicity but also reduces its strength. The healing process of such a bone is also significantly extended as the patient’s cells slowly absorb and turn the transplanted bone into its own; however, often it does not fully disintegrate and there is necrosis of the grafted bone, followed by inflammation [72]. During the first six months after the process of implantation, the nonvascularized cortical grafts become progressively weaker and later resorb. Nevertheless, the area regains structural mechanical strength within twelve months [73]. There are also osteoarticular and osteochondral allografts, of which only in the osteochondral allografts the material is not frozen in order to enable the survival of chondrocytes, which are very sensitive to this process. They are used in joint resurfacing procedures. The osteoarticular allograft is used in arthroplasts, thanks to the fact that a large fragment of bone can be transplanted, which will ensure the efficiency of the joint. This type of allograft is characterized by a deep freeze in order to reduce immunogenicity [74,75]. One further type of allograft is vascularized allograft, but this requires the administration of immunosuppressive drugs to the patient, such as cyclosporine, after surgery. This procedure is performed only in serious cases, such as in large bone defects [72]. This method is associated with many health complications for the patient because they must subsequently take lifelong immunosuppressive drugs that can cause various types of neuropathies, myopathies, and nephropathies. Therefore, the so-called autogenic transplant, i.e., the removal of the patient’s bone from a place of low aesthetic importance, is increasingly used [72]. Although the viability of osteoinductive proteins and osteogenic cells is diminished after such transplantation, it is referred to as the “gold standard” due to the lower risk of infection, transplant rejection, or arthrodesis in the patient. Most often it is taken from the iliac plate (posterior iliac crest); however, it is a very invasive procedure, causing additional complications for the patient, including pain, infection, nerve damage, iatrogenic fracture, incisional hernia, and ending with hematoma [72]. The other sites from which the transplant is performed are the proximal part of the tibia, the distal end of the radius, the distal part of the tibia, and the greater trochanter [72]. Approximately three cubic centimeters of cancellous or corticocancellous graft can be obtained from the distal end of the radius for applications in the hand and upper extremity surgery [40,76,77,78,79]. The greater trochanter can also be used as a useful source of bone graft for surgery in the ipsilateral lower extremity [80]. A cancellous autograft is the best material for filling a bone defect smaller than 6 cm caused by, e.g., cancer or acute bone fracture, allowing it to heal properly without defects. Another example is the nonvascularized cortical autograft, which is used to increase the bone’s structural strength, treating defects up to 6 cm. It is usually performed by cutting out large amounts of the anterior or posterior iliac crest. This way, a large number of osteoprogenitor cells is obtained. Nonvascularized cortical grafts have less mechanical strength several months after transplantation because they are associated with a revascularization process that resorbs parts of the bone to form new blood vessels within the fracture—a process that takes much longer in the cortex than in the trabecular bone. Therefore, they are not recommended for filling cavities larger than 6 cm. Vascularized autografts heal much faster due to the fact that there is no revascularization process in them. It was observed by Waitayawinyu et al. [81] that clinical outcomes for scaphoid non-unions with avascular necrosis improved. Union rates of 93% were observed in those patients who received vascularized grafts. Merrell et al. [82] found in the process of meta-analysis that a vascularized graft may be beneficial for those patients who suffer scaphoid avascular necrosis. Additionally, patients after implantation of vascularized bone graft showed a union rate of 88%. Simultaneously, the recipients of screw-and-wedge fixation achieved a union only 47% of the time. Typically, 90% of osteocytes present in these grafts are able to survive the transplant and bring their blood supply [72]. This type of graft material is most often taken from the ribs, fibula, and scapula, and is mainly used to treat osteonecrosis, non-unions of the scaphoid, and Kienböck’s disease (necrosis of the lunate or osteonecrosis of the lunate) [74,81,82]. In the event that the bone defect is greater than 6 cm, the so-called induced membrane technique can be used. It consists of two key steps: The first is the removal of necrotic bone tissue at the site of the damage, and then the polymethylmethacrylate cement spacer (with or without antibiotics) is applied [83,84,85]. After a few weeks, the second stage begins, which consists of removing the spacer and filling the bone defect with an autogenous cancellous bone graft. Then, the surgeon sutures the wound together with the graft and the membrane, which protects the newly transplanted tissue against too-fast resorption [86]. The use of bone substitutes is increasingly noticed, and they are used with two main advantages in mind: an ability to integrate with the regenerating bone and a strength similar to the [87,88] cortical bone. The most commonly used are ceramics or hydroxyapatite cement, which the patient’s bone uses up and replaces with bone tissue [89,90,91]. Recent studies show that patients with transplanted bone substitutes experience less pain, have fewer complications, and are more agile than patients with other types of transplants [92,93]. In addition to transplants, patients also receive special types of drugs—bone morphogenetic proteins. They stimulate cell division, matrix synthesis, and tissue differentiation. In addition, they also stimulate osteoblasts, osteoclasts, and osteoprogenitor cells to the process of osteogenesis. The strongest activities, and the best-researched and available for treatment, are BMP-2 and BMP-7; however, despite their beneficial bone-forming properties, they increase the risk of cancer and diseases of the genito-urinary system [74].

## 3. Three-Dimensional Printing and Computer-Aided Design 

Three-dimensional (3D) printing, a part of additive manufacturing (AM) and rapid prototyping methods, is a process of creating a 3D object from layer-by-layer-joined material. This makes it an opposite technique to traditional machining, where material is removed from a block to form the desired shape. It is a versatile technique, allowing the fabrication of complex parts from a variety of types of materials. These include polymers, ceramics, metals, and composites. The method can be customized to create various shapes and dense or macro/microporous architecture [94,95,96,97,98,99,100]. Three-dimensional printed objects are being used in many industries. The typical applications include the manufacturing of complex geometries, such as turbine blades, jewelry, molds, implants, prosthetics, mechanical parts, and it is even used in the construction of buildings, tissue engineering, etc. [101,102,103,104,105]. Mentions of the 3D printing process can be found in the late 19th century. These first methods were used when photosculpture and geomorphology technologies were developed. Significant progress was made between the 1980s and 2010s. At that time, a number of 3D printing techniques were developed, including Stereolithography, Fused Deposition Modeling (FDM), ink-jet 3D printing, adhesive-droplet- and powder-bed-based AM, Selective Laser Sintering (SLS), Selective Laser Melting, Continuous Liquid Interface Production, Digital Laser Processing, etc. [106,107,108,109,110,111,112,113,114,115,116,117].

Stereolithography is the first 3D printing process. It uses photocurable polymers that are solidified by a UV laser beam a delivered through a tunable optical system with moving mirrors. The method produces high-level detail but is slow. Digital Light Processing is similar to Stereolithography, as both use light to cure a resin. The difference is that in this case the light passes through a liquid crystal display screen, and the whole layer can be produced at once. This makes the method significantly faster. PolyJet is a method that also uses photocurable resins, but the head of the machine can deliver microdrops of materials of different properties to different spots in the print. Selective Laser Sintering and Multi-Jet Fusion use different approaches to binding polymer powders. The first one uses heat delivered by a laser, while the second one binds the powder using a binding agent. Fused Deposition Modeling uses a heated nozzle to melt material and deposit it on the bed of the machine, and it can print using various materials in the form of spools, granules, or liquids.

Technologies that allow printing from metal powders include Direct Metal Laser Sintering, Electron Beam Melting, Selective Metal Melting, and Selective Laser Sintering. They all heat the material in certain points to induce its fusion. DMLS and SLS use a laser beam but only sinter the powder, which in turn results in a print that should be later heated in an external oven to ensure the proper binding of granules. EBM and SLM methods produce objects made from melted powder. The first method uses an electron beam focused by a series of coils, while the second one is based on a high-power and focused laser. The resulting prints require less postprocessing and are stronger, more uniform, and more robust. Comparison of the methods is presented in Table 1.

Parts created by the use of different technologies have vastly different properties and uses (Table 2). Additionally, printed models can be used as a step in the process of creating an object by casting. Forms can be printed directly, or the material can be melted by hot material poured into the mold, leaving a cavity in the shape of the desired object. 

**Figure 2:**
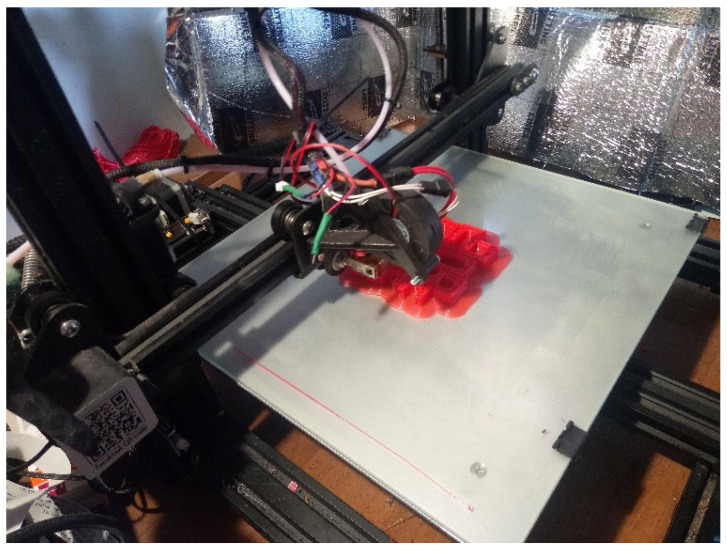
Typical FDM printer during operation. The heated nozzle deposits the material (red) and places it layer by layer on the print bed in the required shape.

Although multiple printing technologies exist, FDM printing is by far the most popular due to its ease of use, the low price of various printing materials with different properties, and the availability of devices and parts (Figure 2). These materials include easy to use and cheap polylactic acid (PLA) and acrylonitrile butadiene styrene (ABS), tough and resistant Polyethylene Terephthalate (PET) and nylon, flexible and shock-absorbing thermoplastic polyurethane (TPU) and thermoplastic elastomer (TPE), water-soluble polyvinyl alcohol (PVA) and other materials doped with carbon for electric conductivity, Kevlar for additional strength, and even metal or wood particles for various looks and applications. Some printers can print using various filaments during the same process using multiple nozzles, making the technology very flexible [139].

**Figure 3:**
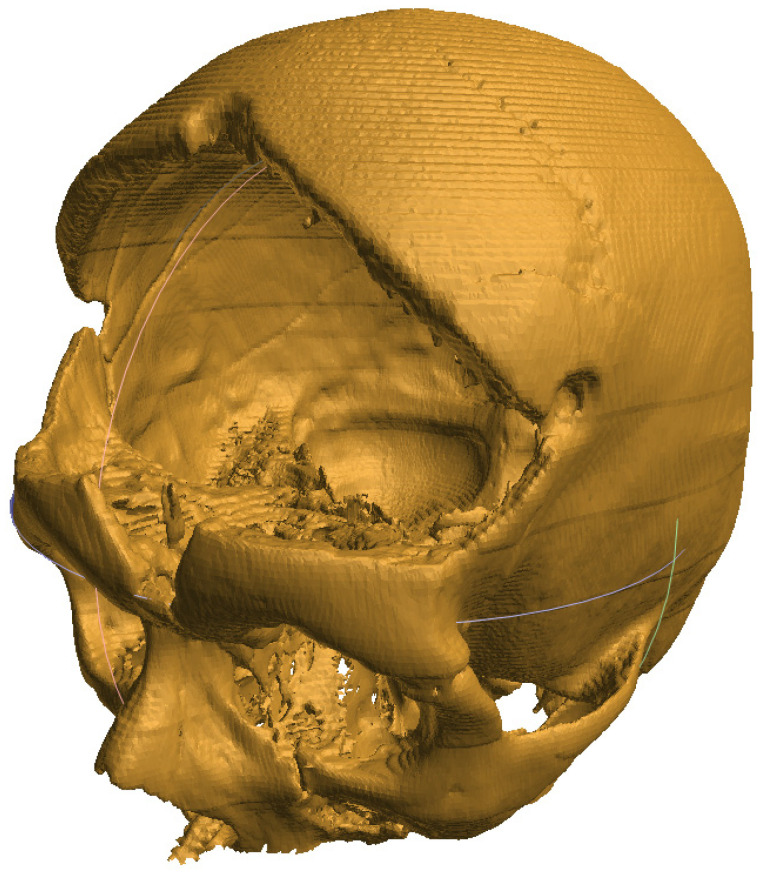
Image of a skull after removal of damaged bone following an accident, obtained by processing a set of tomography images.

**Figure 4:**
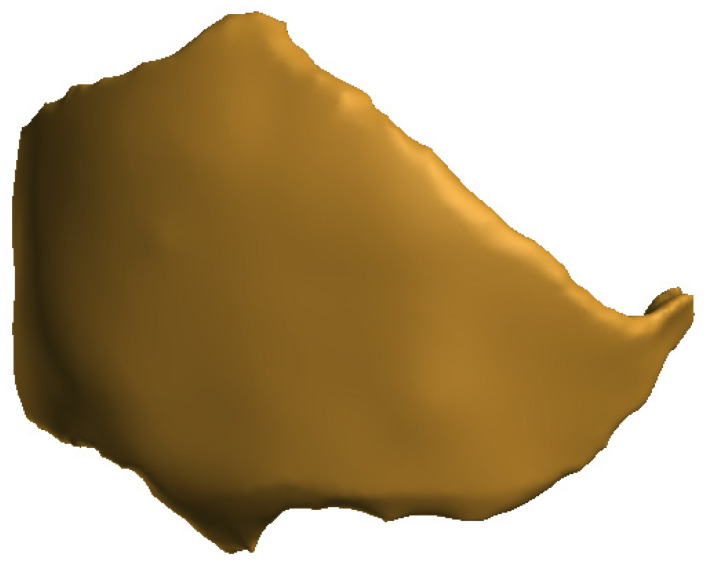
A model of an implant designed to replace the missing bone tissue.

Three-dimensional technology enables the production of implants, tools, instruments, and devices. Now, it is also successfully used in bone tissue engineering to replace critical bone defects [5]. A high-performance scaffold underpins the success of a bone tissue engineering strategy, which is a crucial part of the process. The resulting scaffold should be designed with multiple characteristics in mind, including a desirable shape; physical, chemical, structural, and biological features for regenerating complex bone tissues; and enhanced biological performance [49,140]. Magnetic resonance imaging (MRI) and computed tomography (CT) [141,142] provide images of specific defects in an individual patient, and the images can in turn be further used for 3D printing the defective object [11] (Figure 3 and Figure 4). 

**Figure 5:**
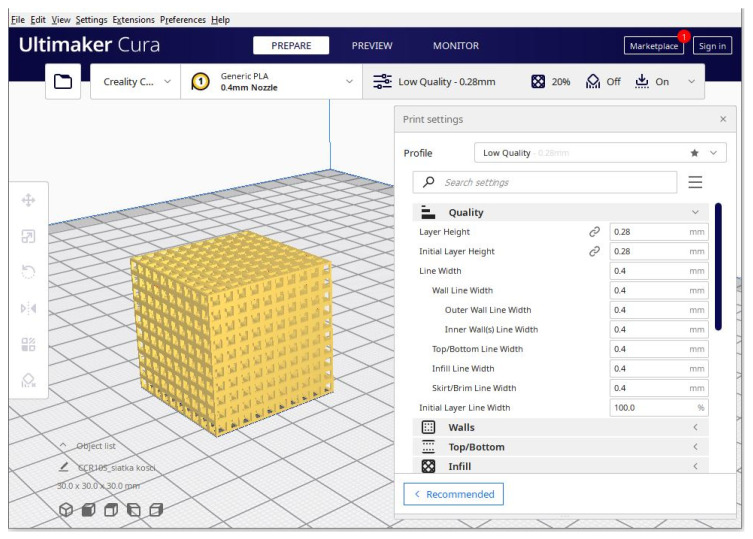
A model of a 30 mm × 30 mm × 30 mm lattice during preprocessing in Ultimaker Cura. The software allows the user to adjust printing parameters for the desired effect.

**Figure 6:**
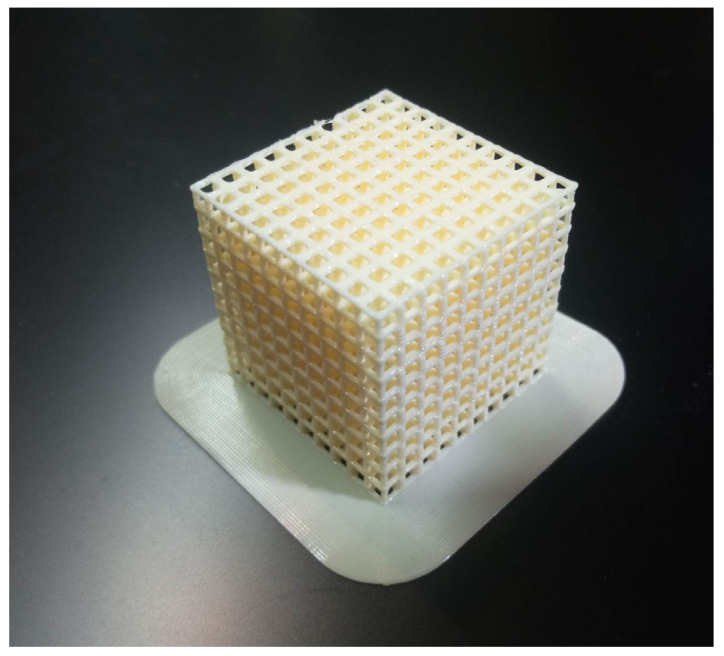
A model of a 30 mm × 30 mm × 30 mm lattice after printing on an FDM printer using PLA.

**Figure 7:**
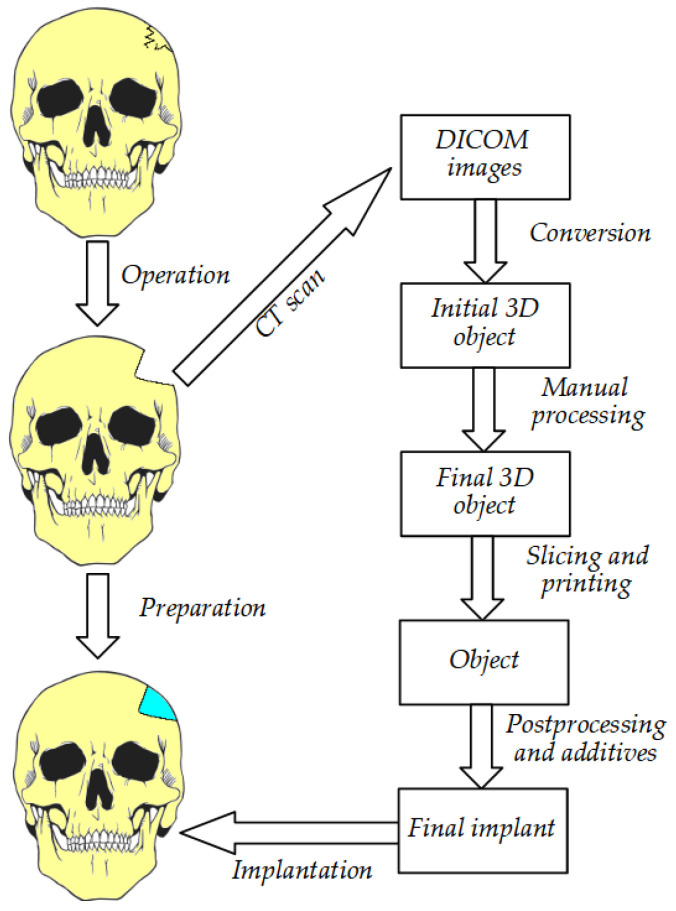
The steps required before final implantation of the prepared structure.

The process of implant preparation can be divided into several steps. The initial stage is collecting data on the defective bone using CT and MRI [141,142]. Through programs such as 3D doctors, 3D slicer, etc., the data are converted into 3D CAD. This is later used as a base for implant creation, as the resulting object must fit exactly in the area in question. This requires the use of graphic design tools and can vary between cases. Currently, the process of model creation is simplified and can be performed using easy to use and free software that creates models that need very little processing before slicing and printing [16]. Nevertheless, the automated algorithms can sometimes struggle when processing complex, low-quality, or noisy images. The skull, especially when damaged in the face area, can be quite challenging and requires a significant amount of manual postprocessing [143]. After the process of computer-aided design is complete, the precise scaffolding design is created [144,145]. During production, scaffolding requires a precise shape, size, and mechanical and biological properties to improve the reliability of patient outcomes after surgery [11,146]. Later, the digital CAD data are processed for 3D printing in an appropriate format and printed with the required material [30] (Figure 5 and Figure 6). Finally, the printed scaffold is planted with cells and tissues and then implanted into the patient’s body during surgery (Figure 7).

Bone tissue engineering with the use of 3D printing provides new insights into the treatment of bone defects thanks to the production of a porous scaffold with appropriate mechanical strength and favorable macro-and microstructures. Tissue function is restored by the appropriate use of cells placed on the scaffolds produced from a combination of biomaterials. The process of healing and osteointegration is heavily influenced by the design of 3D structures, constituting the template for cell transplantation. Multiple parameters must be taken into consideration to ensure the proper performance of scaffolds. These include pore volume, pore size, chemical properties, and mechanical strength. The biomaterials used for scaffold formations need to be structurally stable, fast-curing, biomimetic, biocompatible, and resistant, and have mechanical properties similar to bone [147]. Bones have varying density and elasticity depending on the area and type of the missing piece, and the method of biostructure production must also take that into consideration [3]. For the production of porous bone scaffolds, widely used methods include chemical foaming, freeze drying, solvent casting, and foaming gel [45,47,148,149]. These technologies use materials such as gypsum powder, plastics, resins, aluminide, ceramics, sand, metal, Polyether ether ketone (PEEK), and graphene to create the required part [45]. Appropriate macro- and microstructures are key features in bone tissue engineering scaffolds [49]. Patterned macropores in scaffolds influence cell penetration and cell distribution and, most importantly, enable the transportation of gases and nutrients into the deeper layer of scaffolds, hence maintaining cell viability at a high level [62]. Bioceramic powders, non-hydrogel polymers, natural/synthetic hydrogels, and composites of various materials can be used to formulate printing “inks” for 3D printing. Biodegradable and biocompatible polyesters, such as poly (L-lactic acid) (PLLA), poly-β-hydroxybutyrate (PHB), poly (vinyl alcohol) (PVA), polyurethane elastomers, poly (D, L-lactic acid) (PDLLA), poly (3-hydroxybutyrate-co-3-hydroxyvalerate) (PHBV), polycaprolactone (PCL), poly (lactic-co-glycolic acid) (PLGA) and polyurethanes, can be processed into the form of wires, granules, and even powders. This enables the 3D printing of polymer scaffolds using melting extrusion at high temperature and sintering or dissolving in organic/water solvents in order to enable 3D printing based on micro-extrusion at room/low temperature [62]. The usage of hydrogels allows the trapping of proteins or cells inside the mesh, as well as control over the release of materials as per requirements. In addition, hydrogels due to their absorbable nature and excellent integration capabilities into the surrounding tissues do not need a complex process of surgical removal and also reduce the risk of an inflammatory reaction. Because of their high water-holding capacity (similar to that of soft tissues), hydrogels can support cells better than other 3D scaffolds [150].

**Figure 8:**
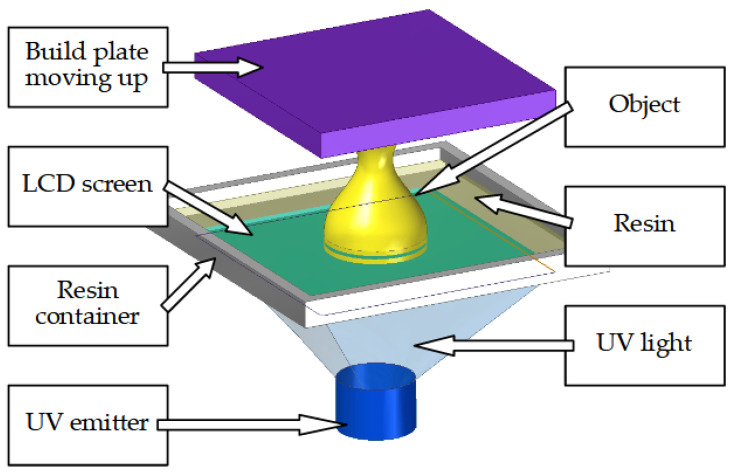
Main components of an SLA printer.

Stereolithography (SLA) is one of the earliest 3D printing techniques used in bone engineering [9]. In this method, the object is created from photosensitive fluid resin. Hardening occurs when resin is exposed to precisely controlled light beams. The process occurs layer by layer and the machine requires few moving parts as the light source is often in the form of an LCD screen and the object is rapidly created one full layer at a time (Figure 8). The method offers high accuracy in the micro- and nanometric scales. It allows the creation of high-resolution complex shapes with an internal architecture. SLA has low biodegradability and biocompatibility. Photo-cross-linkable poly (propylene fumarate) (PPF) is commonly used in SLA [9,151]; however, many other materials such as poly (caprolactone) have also been used. 

However, difficulties have been encountered, one of which is SLA’s inability to print micron-sized scaffolds due to the limitation of the layer thickness and too much cure, which can cause the resin to polymerize in the lower layers. The second difficulty is that there are too few bone engineering materials compatible with SLA due to reduced viscosity, stability, and refractive index. There is also a risk of a cytotoxic effect on enveloped cells due to their radiation by ultraviolet light. Additionally, improper printing parameters can result in the resin not bonding properly. Third, it has also been shown that the size of the light pixels in some SLA processes also limits in-plane microstructure creation [9]. The models created using this method have mediocre mechanical stability and can deteriorate over time. 

**Figure 9:**
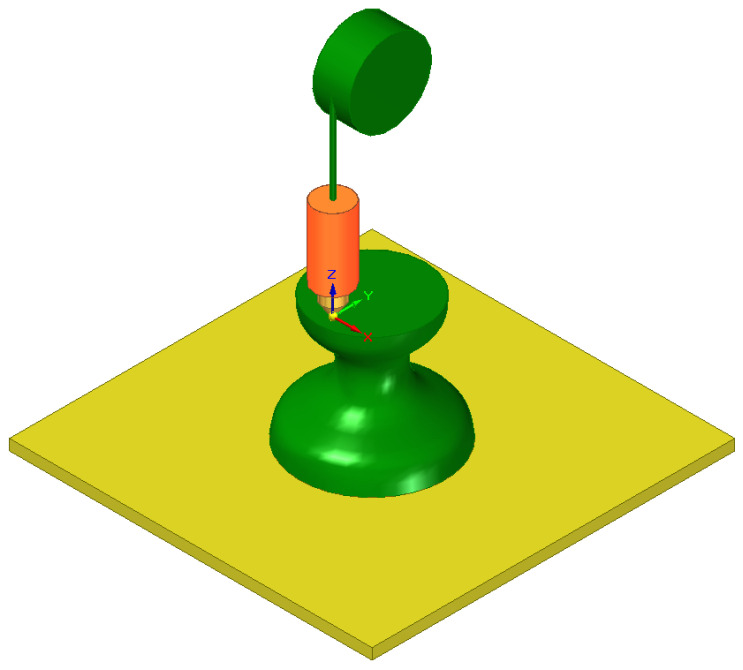
Process of creating an object using FDM.

Fused Deposition Modeling (FDM) is a 3D printing process based on a continuous filament extrusion approach. It allows the fabrication of complex, three-dimensional geometries. The process works by the extrusion of small beads or polymer filament through a small, motorized nozzle in a molten form (Figure 9). The material then hardens post-printing to form a solid construct [9,152]. With this method, the size of the pores in the scaffolds, the morphology, and the joints can be controlled. As a result, the FDM process allows the creation of complex 3D structures that are unattainable in traditional methods such as lithography and micro-machining. Thermoplastic materials, such as PCL, poly(lactic acid) (PLA), and PLGA, are often also combined with other biomaterials, and have been used with FDM to create biocompatible, tissue-engineered scaffolds with a low melting temperature [9]. FDM scaffolds showed favorable mechanical and biochemical properties in the bone regeneration process. The performance of PCL scaffolds in the compression and biocompatibility module has also been demonstrated [153]. On the other hand, PLA scaffolds with different pore sizes showed the appropriate mechanical properties and the distribution of bone marrow stromal cells. The resolution of the FDM printers largely depends on the diameter of the extrusion nozzle. These typically range from 0.3 to 0.8 mm, but in some cases even 0.1 mm or 1 mm can be used [4]. Some studies also show that objects obtained by using FDM printing are sterilized as a result of the high temperatures present in the nozzle [154]. This is a significant positive factor that should be considered when choosing the technology for implant creation. 

Despite its obvious advantages, FDM printing is not without problems. The main limitation of this method is that this technique only produces biological scaffolds with organic shapes and regular structures due to its resolution compared with SLA, as the viscosity of the polymer melts limits the achievable print resolution [9]. Additionally, using a very small nozzle significantly increases printing time and requires really precise calibration and high-quality material, as tiny dust particles can easily clog the opening. Another disadvantage is that popular FDM machines do not support the simultaneous integration of temperature-sensitive cells or biological factors due to the relatively high temperatures used, although it is possible to modify existing designs for such purposes [155]. The overall conversion, processing, and printing time can be long depending on multiple parameters, such as object complexity, nozzle size, the speed of the printer, etc. In some cases, prints may range from several hours to a full day. The method also produces non-uniform objects, as the layers are arranged horizontally. This results in an object that can effectively handle splitting forces perpendicular to the median and frontal planes, while also being weak to splitting across the horizontal plane. This can be slightly remedied by heating the object in a controlled manner to achieve better bonding between the layers. Such an operation can be performed in an open-air environment or after filling the cavities with a powdery medium that is resistant to temperatures, for example table salt or very fine sand. This ensures the dimensional stability of the scaffold when the material is close to its glassing or melting point [156]. After the process, the interlayer bonding is significantly better, leading to increased tensile strength. Additionally, FDM prints often require support material while printing overhangs or at an angle. This material later needs to be removed, either by mechanical processing or by dissolving if using water-soluble materials. The process of design, printing, and processing requires significant technical expertise and experience. Computer-aided design (CAD) tools may be easy to learn but need time to master. The cases can wildly differ from patient to patient, and the designer needs to develop a significant amount of skills and knowledge in medicine, material science, and the practical aspects of printing technologies. Comparison between various grafting techniques is presented in Table 3.

Additive manufacturing by 3D printing offers many possibilities, both for current clinical practice and for the future of the medical industry due to complete freedom of shape design based on patient-specific data. Rapid development in the field of material science will undoubtedly lead to discovering multiple new biocompatible polymers for use in many branches of healthcare. These include, among others, orthopedics, dentistry, prosthetics, and medical simulation. Medical tool production can also be significantly improved by using rapid prototyping. Bioprinting development will allow the production of tissue that can be used to replace the elements of body parts lost in accidents or that are removed during surgery due to various pathologies, fractures, and deformities. This innovative form of therapy will significantly decrease the waiting time of the patients for the procedure and minimize the risk connected to performing allograft and autograft transplantations. In some cases, the usage of 3D printing will benefit patients with conditions that were not curable before. Nevertheless, new technologies also come with new challenges, and this case is no different. The risk involved in all the procedures needs to be assessed, and new guidelines should be put in place to avoid danger to the patients. In the future, we can expect a significant growth in interest regarding the applications of 3D printing in medicine. 

## 4. Conclusions

Bone transplants are associated with the risk of too-turbulent immune reaction, which may even lead to the rejection of the transplant, as well as inflammatory reaction. The preparation for this treatment is long and very complicated in terms of technical difficulty. It is not always possible to perform a bone transplant, which may be associated with chronic pain and chronic inflammation, resulting in amputation. Three-dimensional printing gives provides control over the entire process, including the possibility of creating non-standard shapes and the production of structures with specific physical properties. 

During bone transplantation, the patient can be infected by HIV or HCV. Gamma rays are most commonly used to neutralize these viruses; however, unfortunately, they weaken the grafted material. Three-dimensional printing minimizes the chances of contamination.

The possibility of adjusting the porosity of the grafted fragment through 3D printing allows for the acceleration of the formation of new bone tissue, which has a positive effect on wound healing.

Hydrogels used in 3D printing show excellent integration with surrounding tissues, avoiding the complexity of surgical removal and a reduction in the possibility of an inflammatory reaction. Bone tissue engineering is an innovative approach that can be directly used to repair bone defects during the process of transplantation. During the construction of bone tissue implants, biomaterials play an important role in supporting the regeneration of cells and tissue. We have years of research ahead of us; however, it is certain that 3D printing is the future of transplant medicine. 

## Figures and Tables

**Table 1:** Advantages and disadvantages of various 3D printing technologies.

Type	Technology	Materials	Advantages	Disadvantages	Resolution
Polymer printing	Stereolithography (SLA) [118,119]	UV-curable resins and photopolymers	High level of detail, smooth finish, and tight tolerances	Slow and can be brittle	30 to 140 microns
	Digital Light Processing (DLP) [118,119]	UV-curable resins and photopolymers	High level of detail, smooth finish, and tight tolerances	Fast, suitable for low-volume production, and can be brittle	35 to 100 microns
	Selective Laser Sintering (SLS) [107,120,121]	Nylon-based polymer powders and ceramics	Strong parts and no support structure required	Rough surface finish and slow	80 microns
	Multi-Jet Fusion (MJF) [122,123]	Polymer powders	Strength and fast speed	Rough surface	1200 dpi and 22 microns
	PolyJet [124,125]	Multiple materials	Multiple materials in one print, and colorful prints	Mediocre rigidity	14 microns
	Fused Deposition Modeling (FDM) [119,125]	Multiple polymers with additives	Cost effective, quick, simple, cheap, and many materials	Rough surface finish, slow, and mediocre precision	10 microns vertical; 100 microns horizontal
Metal printing	Direct Metal Laser Sintering (DMLS) [126,127]	Alloy powders	Strong; dense parts	Often requires post processing via sintering and normalizing in a furnace; expensive; rough surface	40 microns
	Electron Beam Melting (EBM) [128,129]	Metal powders	Very strong parts, high speed, energy efficiency, and low distortion	Very expensive	50 microns
	Selective Metal Melting (SLM) [130,131]	Metal powders	Very strong parts; usage of single-component metals	Often requires postprocessing via normalizing in a furnace; expensive	30 microns
	Selective Laser Sintering (SLS) [121,130]	Alumide (aluminum plus polyamide)	Strong parts; no support structure required	Rough surface finish; slow	80 microns
Bioimplant production	Bioprinting [132,133,134,135]	Gels containing living cells and collagen, gelatin, hyaluronan, etc.	Can produce element or complete organs	Young technology	100 microns
Construction 3D printing	Extruding [136,137,138]	Concrete, clay, and soil	Rapid construction of buildings in various shapes, requiring less labor and resulting in less construction waste	Very expensive, large, and complex machines	6000 microns

**Table 2:** Uses of various 3D printing technologies.

Technology	Anatomical Models	Implants	Prosthetics	Dentistry	Industrial Production
Stereolithography (SLA)	yes				
Digital Light Processing (DLP)	yes				yes
Selective Laser Sintering (SLS) polymer	yes		yes		
Multi-Jet Fusion (MJF)	yes				
PolyJet	yes				
Fused Deposition Modeling (FDM)	yes	yes	yes		
Direct Metal Laser Sintering (DMLS)					yes
Electron Beam Melting (EBM)		yes	yes	yes	yes
Selective Metal Melting (SLM)		yes	yes	yes	
Selective Laser Sintering (SLS) metal		yes	yes	yes	

**Table 3:** Comparison between various grafting techniques.

Method		Pros	Cons
Autograft	Cancellous	Biocompatibility Best for defects smaller than 6 cm Fastest healing	Poor availability Disorders at the site of collection
Allograft	Demineralized bone matrix graft	Higher availability Sterilization process	Lower acceptance Lower structural integrity Risk of rejection
	Cancellous	Availability Ease of application No prior harvesting required	Possibility of infection Low initial strength Risk of rejection
	Nonvascular cortical	High initial density of bone Mechanical properties	Weakening of graft after a time Risk of rejection
	Vascularized	Can be used in serious cases	Requires lifelong immunosuppressive drugs High risk of rejection
Synthetic	Ceramic	Rapid resorption and osteointegration Tailored shape and composition	Mediocre mechanical properties Material degradation
	3D printing using SLA	Precisely designed shapes High precision High speed Cheap materials	Lower biocompatibility Degradation over time Mediocre mechanical properties
	3D printing using FDM	Precisely designed shapes Wide range of materials Sterilization during the process High biocompatibility Dimensional stability Cheap materials	Lower print speed than SLA Lower resolution than SLA Non-uniform tensile strength

## Data Availability

No new data was created.
